# Mechanisms of change for two brief alcohol interventions: Testing theoretical mediators for counter attitudinal advocacy and personalized feedback intervention effects

**DOI:** 10.1111/acer.70213

**Published:** 2025-12-04

**Authors:** Angelo M. DiBello, Clayton Neighbors, Melissa R. Hatch, Andrew Weinstein, Kate B. Carey

**Affiliations:** ^1^ Center for Alcohol and Substance Use Studies & Graduate School of Applied and Professional Psychology Rutgers University Piscataway New Jersey USA; ^2^ Department of Psychology University of Houston Houston Texas USA; ^3^ Department of Behavioral and Social Sciences Brown University School of Public Health Providence Rhode Island USA

**Keywords:** alcohol, attitudes, college students, intervention

## Abstract

**Background:**

Given the importance of identifying mechanisms of action for the development and dissemination of alcohol interventions, this study tests theory‐based mechanisms of change for two brief alcohol interventions.

**Methods:**

We conducted a secondary analysis of data from an efficacy trial that compared a novel intervention based on Counter Attitudinal Advocacy (CAA) to an evidence‐based intervention using Personalized Normative Feedback (PNF) and an assessment‐only control. Participants consisted of 585 heavy‐drinking college students who reported experiencing alcohol‐related consequences. Hypothesized mediators were linked to the theoretical underpinning of each intervention: perceived descriptive norms (PNF), dissonance (PNF and CAA), attitudes (CAA), and protective behavioral strategies (CAA). Negative binomial multilevel mediation analyses included data from baseline, posttest, and 1‐, 3‐, and 6‐month follow‐up assessments.

**Results:**

Mediation analyses indicated that, with respect to drinks per week, PNF significantly reduced perceived norms compared to both the control and CAA conditions, which in turn were associated with decreased alcohol consumption. Similarly, CAA significantly reduced dissonance relative to both control and PNF, which was also associated with reduced drinking. Conversely, PNF increased dissonance relative to control, leading to greater alcohol consumption. Parallel patterns emerged for alcohol‐related consequences: PNF reduced norms and CAA reduced dissonance, each associated with fewer consequences, whereas PNF increased dissonance contributing to greater alcohol‐related consequences.

**Conclusions:**

Overall, these findings demonstrate that PNF and CAA operate through distinct mechanisms, emphasizing the complexity inherent in intervention effects. They further highlight the importance of empirically identifying and examining the processes underlying the efficacy of alcohol‐related interventions.

## Graphical abstract

This study identified distinct mechanisms of change for two brief alcohol interventions. Personalized Normative Feedback reduced drinking and consequences by lowering perceived norms, whereas Counter Attitudinal Advocacy reduced both by decreasing dissonance. Findings highlight that even effective interventions can have complex, opposing mechanisms, underscoring the need to empirically test theoretical processes in alcohol intervention research.

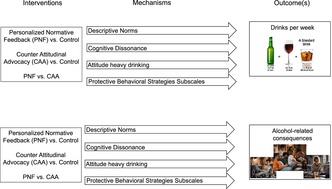

## INTRODUCTION

College alcohol use and misuse remains a significant public health concern. In fact, many aspects of the college environment encourage consuming high quantities of alcohol per occasion (Merrill & Carey, 2016). In 2022, 81% of college students reported drinking in the past year, and 63% reported consuming alcohol in the past month (Patrick et al., 2023). Many of these college students drink heavily, with 28% reporting heavy drinking (5 or more drinks) and 5% reporting high‐intensity drinking (10 or more drinks) in the past 2 weeks (Patrick et al., 2023). A recent study of 19‐ and 20‐year‐old college students found that 56% of drinkers reported at least one alcohol‐related negative consequence in the past year (Patrick et al., 2020), which includes issues such as alcohol poisoning, accidents, blackouts, and sexual assault. Furthermore, this normalization of drinking culture in the college environment can obscure the severity of these risks, making intervention and prevention efforts both urgent and challenging. Addressing this issue requires a comprehensive understanding of the tailored intervention strategies that are used to mitigate its impact on students.

In order to address the pervasive issue of college drinking, a variety of preventive interventions have been developed and implemented across campuses, as summarized in the College Alcohol Intervention Matrix or College AIM (National Institute of Alcohol Abuse and Alcoholism [NIAAA], 2019), while new interventions continue to be developed. Understanding the mechanisms of action for specific interventions is vitally important as they are critical to the advancement of prevention/intervention work. Consistent with the principles of the updated stage model (Onken et al., 2014), attention to mechanisms of action throughout the stages of development facilitates the refinement and optimization of interventions and, ultimately, their implementation in the real world.

This study outlines the theoretical mechanisms of action for two different brief alcohol interventions and tests the hypothesized mediational pathways. The parent study is a recently completed longitudinal randomized controlled trial (RCT) that tested the efficacy of a novel brief alcohol intervention (Counter Attitudinal Advocacy, CAA) relative to an established brief alcohol intervention (Personalized Normative Feedback, PNF) and an assessment‐only control (Carey et al., 2025). The parent RCT recruited 585 students who reported both heavy episodic drinking and at least two alcohol‐related negative consequences in the past month. Participants were randomly assigned to one of the three conditions (CAA, PNF, control), and follow‐up assessments occurred at 1, 3, and 6 months postintervention. Each active intervention was based on a distinct theoretical mechanism of action, described next.

### Interventions within the current study

#### Personalized normative feedback (PNF)

As summarized in College AIM, normative feedback to correct misperceived descriptive norms has become a prominent change strategy supported by many college drinking intervention studies (NIAAA, 2019); normative feedback has been incorporated into multicomponent interventions often in the form of personalized normative feedback (PNF; e.g., Dimeff et al., 1999). Evidence from several reviews of RCTs suggests that stand‐alone computer‐delivered PNF is effective in reducing drinking among high‐risk students (Cronce & Larimer, 2011; Dotson et al., 2015), especially when administered in a supervised setting (Rodriguez et al., 2015). The PNF administered in the parent study was associated with significantly fewer drinks per week relative to the assessment‐only control (Carey et al., 2025). The purported mechanism of change—reductions in exaggerated perceptions of how much/often other students drink—has received substantial support in prior studies (Reid & Carey, 2015). Despite the promise of PNF, it is not without its limitations; specifically, for some individuals, standard PNF paradigms can evoke defensiveness (e.g., Nye et al., 1999) and reactance among participants, which can lead to smaller reductions in alcohol use (Boyle et al., 2018; Jung et al., 2010). Within the present work, we aimed to replicate existing findings by again examining changes in perceptions of descriptive norms as a key mechanism of change specific to PNF on drinking outcomes.

#### Counter attitudinal advocacy (CAA)

Recent work has shown that counter‐attitudinal advocacy (Festinger & Carlsmith, 1959) has demonstrated promise when adapted for alcohol prevention (Carey et al., 2025; DiBello, Carey, & Cushing, 2018a). The CAA task prompts participants to advocate for a position contrary to an existing attitude or behavior, an activity designed to evoke cognitive dissonance. Dissonance may be reduced by changing future behavior or attitudes. CAA paradigms require that a participant voluntarily place him/herself in a public position contrary to his or her existing beliefs or behavior via exercises such as video recording (Simmons et al., 2013) or speech delivery (Simmons et al., 2004; Simmons & Brandon, 2007). Across a range of behaviors, CAA‐induced dissonance has produced changes in attitudes, behavioral intentions, and actual behaviors (Kim et al., 2014). While this paradigm has evidenced preliminary efficacy with regard to drinking outcomes, including a significant reduction in alcohol‐related consequences in the parent study (Carey et al., 2025), there have been no tests of mediation specific to CAA and alcohol use. Thus, the current work aimed to explore theory‐based mechanisms that could explain CAA's efficacy.

**FIGURE 1:**
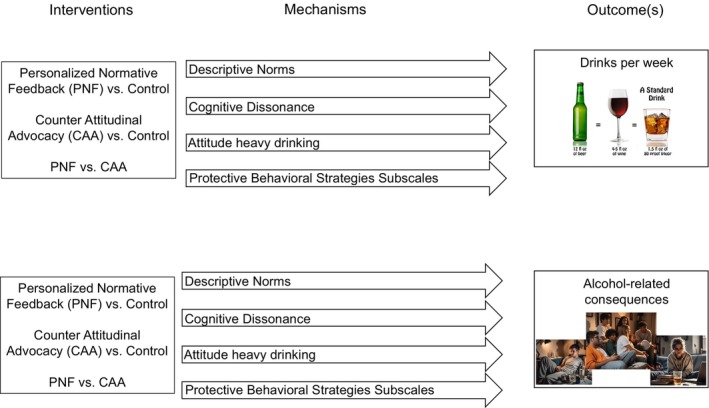
A visual abstract that illustrates the examination of different mechanisms of action for PNF and CAA.

Although PNF and CAA originate from distinct theoretical traditions, both interventions can be understood as targeting discrepant beliefs and thereby evoking dissonance, albeit through different pathways. PNF primarily operates through a cognitive route, correcting exaggerated perceptions of peer drinking norms and prompting behavior change by challenging beliefs and providing socially relevant information to guide behavior. In contrast, CAA relies more heavily on an emotional and motivational route, eliciting cognitive dissonance when participants advocate against their own behaviors or attitudes, which may then motivate shifts in future drinking‐related decisions to reduce the dissonance. Thus, while both interventions share dissonance as a broad underlying principle, they do so through different mechanisms—PNF emphasizing changes in perceived norms and CAA emphasizing dissonance‐driven attitude and behavior change. This contrast underscores the importance of examining intervention‐specific mediators within this study (Figure 1).

### Hypothesized theory‐based mechanisms of CAA

#### Dissonance

Briefly, cognitive dissonance theory suggests that individuals have a drive to hold personal attitudes, beliefs, and behavior in agreement with one another to avoid dissonance (Festinger, 1957). Cognitive tension (i.e., dissonance) can result from attitude–attitude, attitude–behavior, or behavior–behavior discrepancies (Cooper, 2007). Individuals are motivated to reduce or eliminate this discomfort to achieve consonance (i.e., agreement). After publicly endorsing a stance in opposition to one's prior position, the person could adjust their attitudes to align more closely with the advocated position in order to alleviate the dissonance. Alternatively, after publicly endorsing an opposing stance, one may resolve the dissonance by expressing a greater intention to act in accordance with their statement.

While dissonance is often implicated as an important element of PNF and CAA efficacy, no recent mediation tests of this process have been conducted. Indeed, PNF is consistent with the *dissonance‐based belief disconfirmation paradigm* (Festinger et al., 1956), whereby dissonance is aroused when participants are provided with information inconsistent with their beliefs. That is, social norms approaches to alcohol intervention provide individuals with social norm information that is inconsistent with and challenges their beliefs about a variety of drinking behaviors, such as frequency of use, amount consumed, and/or problems experienced. Further, CAA is consistent with the *induced compliance paradigm* (Festinger & Carlsmith, 1959), wherein participants who act contrary to an existing attitude are hypothesized to experience dissonance. This dissonance may be reduced by changing future behavior or attitudes. Therefore, the present work aimed to examine dissonance as a mediator of both the PNF and CAA interventions.

#### Attitudes

Attitudes are favorable or unfavorable predispositions toward an attitude object (in this case, alcohol use resulting in negative consequences), and alcohol‐related attitudes play a central role in CAA. Attitudes represent a prominent component of many different health behavior theoretical models (Montaño & Kasprzyk, 2015), and with regard to alcohol use and associated consequences, attitudes routinely evidence strong predictive utility in longitudinal studies (DiBello et al., 2022, 2025; DiBello, Miller, et al., 2018b). Recent work also suggests that personal attitudes are stronger predictors of alcohol consumption than descriptive norms and injunctive norms (DiBello et al., 2022; DiBello, Miller, et al., 2018b). To date, no strong tests of mediation via attitude change exist in the college alcohol prevention literature (Reid & Carey, 2015), perhaps because neither individualized brief interventions nor mass media (e.g., social norms) campaigns explicitly target attitude change using established methods of changing attitudes. Thus, the current work aimed to examine changes in attitude toward heavy drinking as a mechanism of action specific to CAA.

#### Protective behavioral strategies (PBS)

PBS are specific behaviors individuals can engage in to reduce the negative consequences of alcohol use (Martens et al., 2007) and represent individual‐level harm reduction strategies. Within the CAA condition, participants articulated ways to avoid consequences using self‐generated PBS; we posited that this writing activity would make PBS more salient, leading to an increase in the use of those strategies. Generally, reviews confirm that the use of PBS is associated with fewer alcohol‐related problems (Cox et al., 2024). Indeed, Witkiewitz et al. (2022) suggest that promoting the use of PBS may serve as an effective mechanism of change, particularly for young adult risky drinkers who seek to reduce alcohol‐related harms while continuing to drink. Therefore, writing about self‐generated ways to use PBS during drinking occasions should produce more PBS, which should result in fewer alcohol‐related consequences. One PBS scale, the Protective Behavioral Strategies Scale (PBSS; Martens et al., 2005), is currently the most widely used scale of alcohol PBS and includes three subscales: Stopping/Limiting, Manner of Drinking, and Serious Harm Reduction. The use of certain PBS (e.g., stopping/limiting) is consistently inversely correlated with both alcohol use and alcohol‐related problems (Cox et al., 2024); on the other hand, there are mixed results on the association between serious harm reduction and alcohol outcomes in the literature with some studies demonstrating null relationships or positive associations (see Cox et al., for review). However, there is some work (e.g., Braitman et al., 2015) that demonstrates that the manner in which PBS variables are scored can impact their respective associations with alcohol outcomes as well as context‐specific effects on PBS use that impact the PBS➔alcohol outcome association (Braitman et al., 2017). Therefore, it is possible that the intervention could impact general PBS and not have a subsequent impact on alcohol‐related outcomes. Thus, the present work aimed to examine whether changes in any of the PBS subscales mediated the CAA intervention.

### Current study

This study leverages data from a longitudinal RCT to examine four key mediators—normative perceptions, cognitive dissonance, alcohol‐related attitudes, and PBS—that may explain the efficacy of PNF and CAA in reducing personal alcohol use and related harms among college students. Because these interventions were associated with improvements in drinks per week and alcohol‐related consequences, respectively, we focus on these outcomes. Specific hypotheses are presented as follows.

#### Hypothesis 1: Normative perceptions (PNF‐specific mechanism)

We hypothesize that participants receiving the PNF intervention will exhibit significant reductions in perceptions of peer drinking relative to control participants. In turn, these (presumably more accurate) normative perceptions are expected to mediate decreases in alcohol consumption. Specifically, we anticipate that:

PNF (but not CAA) will lead to lower estimates of peer drinking.

Reductions in perceived descriptive norms will mediate subsequent reductions in drinks per week.

#### Hypothesis 2: Cognitive dissonance (common mechanism for PNF and CAA)

Both interventions were theorized to induce cognitive dissonance—either through providing information that contradicts personal beliefs (PNF) or through requiring public advocacy against one's own behaviors (CAA). We hypothesized that:

Higher levels of postintervention cognitive dissonance would be observed in both intervention groups relative to control.

The degree of induced dissonance would mediate the interventions' effects on drinks per week and alcohol‐related consequences.

#### Hypothesis 3: Alcohol‐related attitudes (CAA‐specific mechanism)

Given that CAA requires participants to advocate for positions contrary to their existing behaviors, we hypothesized that CAA would reduce positive attitudes toward heavy drinking. We expected that:

Participants in the CAA condition would report reductions in positive attitudes toward heavy drinking after the intervention relative to both PNF and control.

The change (reduction) in positive attitudes toward heavy drinking would mediate the relationship between the CAA intervention and reductions in drinks per week and alcohol‐related consequences.

#### Hypothesis 4: Protective behavioral strategies (CAA‐specific mechanism)

Engagement in protective behavioral strategies has been associated with fewer alcohol‐related harms. We proposed that the CAA intervention would encourage greater use of PBS, which would, in turn, mediate reductions in alcohol use and associated consequences. Specifically, we hypothesized that:

Participants receiving the CAA intervention would report increased use of PBS (e.g., strategies for stopping/limiting, modifying drinking behavior) following the intervention relative to both the PNF and control conditions.

Enhanced use of PBS would mediate the association between the CAA intervention condition and drinks per week and alcohol‐related consequences.

Collectively, these hypotheses aimed to elucidate the distinct and shared pathways through which PNF and CAA exert their beneficial effects. Understanding these mediational processes will not only advance theoretical models of behavior change but also inform the refinement of targeted interventions to more effectively reduce problematic alcohol use among college students.

## METHODS

### Participants

A total of 585 heavy‐drinking undergraduate students aged 18–26 from two US universities participated between November 2019 and June 2022. Site A was a private residential medium‐size university in the Northeast, and Site B was a large public commuter university in the South. Eligibility required recent heavy drinking (4+/5+ drinks per occasion for women/men), two or more alcohol‐related problems in the past month, and not participating in other alcohol studies. Participants were not excluded on the basis of using multiple substances (e.g., marijuana, other drugs) in addition to alcohol. After COVID‐19, Site A limited eligibility to students living on or near campus.

### Procedure

Participants were recruited via campus‐specific methods, including emails, flyers, and online postings, with eligibility screened through an online survey. Baseline sessions, initially in‐person (*n* = 110), transitioned to remote delivery via Zoom during COVID‐19 lockdowns (*n* = 475) and remote delivery continued after the return to on‐campus instruction (detailed in Carey et al., 2025). Informed consent was obtained, and participants completed a Qualtrics survey, branching them into one of three conditions: Counter‐Attitudinal Advocacy (CAA), Personalized Normative Feedback (PNF), or an assessment‐only control. Each intervention lasted between 5 and 10 min.

### Intervention conditions

#### CAA

Participants completed a self‐reflective writing activity in response to a written prompt wherein they were asked to describe (a) why drinking in a way that avoids problems was a good idea and (b) self‐generated protective behavioral drinking strategies for avoiding alcohol‐related consequences. Unknown to the participant, the writing prompt included (as examples) consequences the participant had reported experiencing during the baseline survey. They summarized their written response to the RA to add a public aspect to the activity, consistent with the CAA literature. Please see Carey et al. (2025), for more details on the intervention.

#### PNF

Participants reviewed and compared graphical representations of three separate aspects of their drinking patterns (drinking frequency, drinks per occasion, drinks per week). Each graph portrayed their own behavior, next to actual campus‐specific norms for their gender identity, and their perceptions of that same group. Feedback was explained by a research assistant (RA) and provided in hard copy or via email.

#### Assessment‐only control condition

At the end of the baseline survey, participants saw a screen with instructions to contact the RA who was waiting in an outer office or muted on the Zoom call (post‐COVID‐19). No other activities occurred, and the participant was directed to the brief (5–7 min) postintervention survey.

### Postintervention and follow‐ups

Participants received alcohol and mental health resources and $25 compensation for their baseline session. Follow‐up surveys at 1, 3, and 6 months were incentivized with increasing compensation ($25, $30, and $35, respectively) and multiple reminders.

### Measures

Measures were selected to be relevant to and valid for college drinkers and served as (a) sample descriptors, (b) primary outcomes, and (c) mediators.

Participants provided demographic information, including their age, birth sex, gender identity, year in college, and current living situation.

### Primary outcomes

All alcohol items used a 1‐month time frame and standard drink equivalents of 12 oz. of beer, 5 oz. of 12% table wine, 12 oz. of wine cooler, or 1.25 oz. of 80‐proof liquor. Alcohol consumption was measured using the Daily Drinking Questionnaire (Collins et al., 1985), which asks participants to estimate the number of drinks they typically consume each day of the week in the past 30 days. Drinks were summed across days to determine drinks per week.

*Alcohol‐related consequences* in the last month were assessed using the Young Adult Alcohol Consequences Questionnaire (YAACQ; Read et al., 2006), a 48‐item checklist of problems related to drinking that is tailored to college drinkers. Items included “I have passed out from drinking” and “I have taken foolish risks when I have been drinking.” Response options (no/yes) were scored 0 or 1 and were summed to produce a total score ranging from 0 to 48. Alphas in this study ranged from 0.89 to 0.92 across the four assessments.

### Mediators

*Perceived descriptive norms* were derived from a modified version of the Drinking Norms Rating Form (DNRF; Baer et al., 1991). The DNRF assesses perceived typical weekly drinking by having participants fill in the average number of standard drinks they think the typical (male/female) student at their school consumes for each day of the week over the previous month, and responses were summed across days to produce a total descriptive norms score. The DNRF has been used in multiple studies of college drinking and has shown good concurrent and prospective validity (Neighbors et al., 2006).

*Attitudes* toward heavy consumption were assessed with a measure used in previous research (DiBello et al., 2020, 2023; DiBello, Carey, & Cushing, 2018a; DiBello, Miller, et al., 2018b) modified from previous research (Hagger et al., 2012). The stem read, “Having five or more drinks (for males)/four or more drinks (for females) in a sitting over the next month would be…” Both measures use five semantic differential scales that range from 1 to 5: unenjoyable–enjoyable, bad–good, harmful–beneficial, foolish–wise, and unpleasant–pleasant. Alphas in this study ranged from 0.84 to 0.87 across the four assessments.

*Cognitive dissonance* was assessed using the Dissonance Thermometer (Devine et al., 2019; Elliot & Devine, 1994; Simmons et al., 2004, 2013; Simmons & Brandon, 2007), administered in the postintervention survey only. Items include asking participants how uncomfortable, angry at themselves, shameful, uneasy, disgusted with themselves, embarrassed, bothered, annoyed with themselves, and disappointed with themselves they feel immediately following the CAA, PNF, and Control activities. Alpha was 0.94.

*Protective Behavioral Strategies* were measured using the 15‐item Protective Behavioral Strategies Scale (Martens et al., 2005), which contains three subscales: Limiting/Stopping Drinking (7 items; “Determine not to exceed a set number of drinks”), Manner of Drinking (5 items; “Drink slowly, rather than gulp or chug”), and Serious Harm Reduction (3 items; “Use a designated driver”). The PBSS assesses the contingent frequency of using each strategy when drinking (1 = never, 6 = always) and is reliable and valid for use with college drinkers. Alphas in this study ranged from 0.87 to 0.93 across the four assessments for Limiting/Stopping Drinking, 0.89 to 0.92 across the four assessments for Manner of Drinking, and 0.89 to 0.94 across the four assessments for Serious Harm Reduction.

### Analysis plan

The primary purpose of the current manuscript was to use mediation analyses to identify the mechanisms of action for CAA and PNF. The mediation analyses were conducted using a product of coefficients method adapted for nonlinear mediators and outcomes (Geldhof et al., 2018; MacKinnon et al., 2023). Specifically, partial derivatives were used for all paths involving nonlinear associations (Kim & McCabe, 2022).

We utilized a multilevel approach with level‐2 predictors (CAA and PNF), a level‐1 mediator, and level‐1 outcomes. All models for the drinks per week and alcohol‐related consequences outcomes, as well as descriptive norms and dissonance mediators, were modeled as negative binomial distributions. The attitude and PBS mediators were modeled using Gaussian distributions for the a‐paths and negative binomial distributions for the b‐paths. For each mediation model, the coefficients used to estimate the mediation effects were based on two multilevel models (one for the a‐path and one for the b‐path).

For all the models predicting drinks per week, the first models (models predicting the mediators) included baseline values of drinks per week, baseline values of the mediator (for all but dissonance, which was only measured postintervention), time (coded 0, 1, 3, and 6), site, birth sex (male = 1, female = 0), PNF (vs. control), and CAA (vs. control). The second models (predicting changes in drinks per week) included baseline values of drinks per week, as well as baseline and concurrent values of the mediator, time, site, biological sex, PNF (compared to control), and CAA (compared to control). For the alcohol‐related consequences models, the first models (again models predicting the mediators) included baseline values of alcohol‐related consequences, baseline values of the mediator (for all but dissonance, which was only measured postintervention), concurrent values of alcohol consumption, time, site, biological sex, PNF (vs. control), and CAA (vs. control). The second models predicting changes in alcohol‐related consequences all included baseline values of alcohol‐related consequences, baseline and concurrent values of the mediator, concurrent values of alcohol consumption, follow‐up time point, site, biological sex, PNF (vs. control), and CAA (vs. control). Both the alcohol consumption and related consequence models were parallel in structure and included the same covariates as those that were reported in the main outcomes paper (Carey et al., 2025).

In this approach to testing mediation, the *a*‐paths represent the difference in the partial derivatives of the mediator as a function of the condition comparisons. The *b*‐path was represented by the partial derivative for each mediator of interest. Both models included random intercepts. Each indirect effect was estimated based on the procedures recommended by Geldhof et al. (2018). Specifically, when examining the changes in descriptive norms as a mediator of the intervention effect on drinks per week, with both descriptive norms (mediator) and drinks per week (outcome) being modeled using multilevel negative binomial regression, the *a*‐path is represented as the partial derivative of the intervention effect on changes in descriptive norms. The *b*‐path is represented as the partial derivative of the association between changes in descriptive norms and changes in drinks per week. The indirect effects were evaluated as the product of these partial derivatives using percentile‐based bootstrapped confidence intervals with 10,000 replications. The significance level (*α*) was adjusted to account for testing multiple mediators by applying a Bonferroni correction, whereby the conventional significance level (*α* = 0.05) was divided by the number of mediators (6), yielding an adjusted *α* level of 0.008. This adjustment is reflected in the confidence interval range (99.2%) used to determine the statistical significance of indirect effects.

## RESULTS

### Sample description

Participants identified primarily as female (62.8%), with a mean age of 19.9 (SD = 1.6). With regard to ethnicity, 23% identified as Hispanic/Latino, and the sample endorsed the following racial identities: 35.6% non‐Hispanic White, 25.7% Asian, 10.2% non‐Hispanic Black, and 5.4% as other. At baseline, participants reported drinking 9.93 (SD = 6.68) drinks per week on average and reported experiencing 11.54 (SD = 7.30) alcohol‐related consequences in the past 30 days.

### Mediation results

The full mediation results are presented in Table 1 for drinks per week and Table 2 for alcohol‐related consequences. Please see Tables S1–S4, for the complete model results for each outcome and mediator. We evaluated the indirect effects of each comparison (i.e., CAA vs. Control, PNF vs. Control, CAA vs. PNF) on changes in drinking outcomes through changes in each respective mediator.

**TABLE 1:** Mediation of intervention effects on changes in drinks per week.

Outcome: Changes in drinks per week						
Mediator	Path	Predictor	*ab*	SE	LL99.2%	UL99.2%
Descriptive norms	***a*‐path**	**PNF vs. Control**	**−4.686**	**0.203**	**−5.259**	**−4.199**
	*a*‐path	CAA vs. Control	0.297	0.231	−0.322	0.895
	***a*‐path**	**CAA vs. PNF**	**4.983**	**0.210**	**4.464**	**5.585**
	***b*‐path**	**Descriptive Norms**	**1.027**	**0.062**	**0.780**	**1.098**
	**Indirect effect**	**PNF vs. Control**	**−4.814**	**0.360**	**−5.424**	**−3.525**
	Indirect effect	CAA vs. Control	0.305	0.220	−0.308	0.854
	**Indirect effect**	**CAA vs. PNF**	**5.119**	**0.378**	**3.737**	**5.730**
Attitude toward heavy drinking	*a*‐path	PNF vs. Control	−0.048	0.037	−0.141	0.057
	*a*‐path	CAA vs. Control	0.061	0.035	−0.032	0.153
	***a*‐path**	**CAA vs. PNF**	**0.109**	**0.037**	**0.006**	**0.202**
	*b*‐path	Attitude	0.235	0.106	−0.186	0.380
	Indirect effect	PNF vs. Control	−0.011	0.007	−0.034	0.013
	Indirect effect	CAA vs. Control	0.014	0.008	−0.014	0.036
	Indirect effect	CAA vs. PNF	0.026	0.012	−0.021	0.050
Dissonance	***a*‐path**	**PNF vs. Control**	**3.771**	**1.301**	**0.281**	**7.076**
	***a*‐path**	**CAA vs. Control**	**−4.048**	**1.142**	**−7.194**	**−1.069**
	***a*‐path**	**CAA vs. PNF**	**−7.820**	**1.179**	**−11.020**	**−4.713**
	***b*‐path**	**Dissonance**	**1.268**	**0.055**	**1.010**	**1.313**
	**Indirect effect**	**PNF vs. Control**	**4.780**	**1.531**	**0.321**	**8.258**
	**Indirect effect**	**CAA vs. Control**	**−5.131**	**1.344**	**−8.420**	**−1.220**
	**Indirect effect**	**CAA vs. PNF**	**−9.912**	**1.440**	**−13.091**	**−5.427**
PBS—stopping/limiting	***a*‐path**	**PNF vs. Control**	**0.114**	**0.035**	**0.031**	**0.214**
	***a*‐path**	**CAA vs. Control**	**0.141**	**0.034**	**0.052**	**0.235**
	*a*‐path	CAA vs. PNF	0.027	0.035	−0.074	0.112
	*b*‐path	Stopping/Limiting	0.423	0.251	−0.096	1.276
	Indirect effect	PNF vs. Control	0.048	0.038	−0.011	0.194
	Indirect effect	CAA vs. Control	0.060	0.042	−0.011	0.221
	Indirect effect	CAA vs. PNF	0.012	0.022	−0.052	0.089
PBS—manner of drinking	*a*‐path	PNF vs. Control	−0.008	0.044	−0.136	0.100
	*a*‐path	CAA vs. Control	−0.076	0.045	−0.193	0.044
	*a*‐path	CAA vs. PNF	−0.067	0.045	−0.174	0.064
	*b*‐path	Manner of Drinking	0.229	0.194	−0.327	0.714
	Indirect effect	PNF vs. Control	−0.002	0.012	−0.056	0.033
	Indirect effect	CAA vs. Control	−0.017	0.019	−0.088	0.035
	Indirect effect	CAA vs. PNF	−0.015	0.016	−0.079	0.029
PBS—serious harm reduction	*a*‐path	PNF vs. Control	−0.058	0.062	−0.223	0.105
	*a*‐path	CAA vs. Control	−0.087	0.061	−0.258	0.070
	*a*‐path	CAA vs. PNF	−0.028	0.060	−0.195	0.129
	***b*‐path**	**Serious Harm Reduction**	**1.517**	**0.159**	**1.076**	**1.934**
	Indirect effect	PNF vs. Control	−0.089	0.094	−0.350	0.160
	Indirect effect	CAA vs. Control	−0.131	0.092	−0.395	0.111
	Indirect effect	CAA vs. PNF	−0.043	0.091	−0.289	0.198

*Note*: Bold text represents a significant effect.

**TABLE 2:** Mediation of intervention effects on changes in alcohol‐related consequences.

Outcome: Changes in alcohol‐related consequences						
Mediator	Path	Predictor	*ab*	SE	LL99.2%	UL99.2%
Descriptive norms	***a*‐path**	**PNF vs. Control**	**−4.546**	**0.201**	**−5.117**	**−4.068**
	*a*‐path	CAA vs. Control	0.320	0.228	−0.267	0.925
	***a*‐path**	**CAA vs. PNF**	**4.866**	**0.210**	**4.358**	**5.481**
	***b*‐path**	**Descriptive Norms**	**0.841**	**0.077**	**0.491**	**0.898**
	**Indirect effect**	**PNF vs. Control**	**−3.823**	**0.389**	**−4.268**	**−2.202**
	Indirect effect	CAA vs. Control	0.269	0.161	0.191	0.672
	**Indirect effect**	**CAA vs. PNF**	**4.092**	**0.411**	**2.348**	**4.550**
Attitude toward heavy drinking	*a*‐path	PNF vs. Control	−0.013	0.037	−0.115	0.083
	*a*‐path	CAA vs. Control	0.063	0.035	−0.029	0.157
	*a*‐path	CAA vs. PNF	0.077	0.037	−0.015	0.182
	***b*‐path**	**Attitude**	**0.620**	**0.131**	**0.017**	**0.721**
	Indirect effect	PNF vs. Control	−0.008	0.015	−0.053	0.037
	Indirect effect	CAA vs. Control	0.039	0.016	−0.011	0.077
	Indirect effect	CAA vs. PNF	0.048	0.019	−0.006	0.094
Dissonance	***a*‐path**	**PNF vs. Control**	**4.637**	**1.449**	**0.848**	**8.566**
	***a*‐path**	**CAA vs. Control**	**−4.933**	**1.065**	**−7.961**	**−2.234**
	***a*‐path**	**CAA vs. PNF**	**−9.570**	**1.272**	**−12.982**	**−6.410**
	***b*‐path**	**Dissonance**	**0.727**	**0.070**	**0.399**	**0.775**
	**Indirect effect**	**PNF vs. Control**	**3.373**	**0.913**	**0.484**	**5.336**
	**Indirect effect**	**CAA vs. Control**	**−3.588**	**0.730**	**−5.148**	**−1.206**
	**Indirect effect**	**CAA vs. PNF**	**−6.961**	**1.009**	**−8.572**	**−3.327**
PBS—stopping/limiting	***a*‐path**	**PNF vs. Control**	**0.123**	**0.036**	**0.034**	**0.219**
	***a*‐path**	**CAA vs. Control**	**0.143**	**0.034**	**0.053**	**0.235**
	*a*‐path	CAA vs. PNF	0.019	0.035	−0.077	0.111
	*b*‐path	Stopping/Limiting	0.625	0.305	−0.154	1.461
	Indirect effect	PNF vs. Control	0.077	0.046	−0.020	0.230
	Indirect effect	CAA vs. Control	0.089	0.050	−0.021	0.249
	Indirect effect	CAA vs. PNF	0.012	0.025	−0.062	0.098
PBS—manner of drinking	*a*‐path	PNF vs. Control	−0.029	0.044	−0.149	0.089
	*a*‐path	CAA vs. Control	−0.073	0.045	−0.190	0.048
	*a*‐path	CAA vs. PNF	−0.044	0.045	−0.162	0.076
	*b*‐path	Manner of Drinking	0.083	0.229	−0.431	0.809
	Indirect effect	PNF vs. Control	−0.002	0.015	−0.064	0.037
	Indirect effect	CAA vs. Control	−0.006	0.021	−0.093	0.044
	Indirect effect	CAA vs. PNF	−0.004	0.016	−0.077	0.035
PBS—serious harm reduction	*a*‐path	PNF vs. Control	0.015	0.061	−0.146	0.178
	*a*‐path	CAA vs. Control	−0.078	0.060	−0.238	0.082
	*a*‐path	CAA vs. PNF	−0.093	0.059	−0.250	0.058
	***b*‐path**	**Serious Harm Reduction**	**1.388**	**0.192**	**0.969**	**1.987**
	Indirect effect	PNF vs. Control	0.021	0.091	−0.222	0.273
	Indirect effect	CAA vs. Control	−0.108	0.088	−0.368	0.122
	Indirect effect	CAA vs. PNF	−0.130	0.088	−0.390	0.085

*Note*: Bold text represents a significant effect.

For drinks per week, tests of *b*‐paths between mediators and outcome were significant for descriptive norms, dissonance, and serious harm reduction, but not significant for attitudes toward heavy drinking, stopping/limiting PBS, or manner of drinking PBS. The mediation results show several significant indirect effects, as follows. PNF reduced norms compared to both control and CAA, which was associated with fewer drinks per week. CAA reduced dissonance compared to both control and PNF, which was associated with fewer drinks per week. In contrast, PNF increased dissonance relative to control, which was associated with more drinks per week. Also, PNF and CAA both increased stopping/limiting PBS, but this was not associated with changes in drinks per week.

For alcohol‐related consequences, *b*‐paths between mediators and outcomes were significant for descriptive norms, attitude, dissonance, and serious harm reduction, but not significant for stopping/limiting PBS, or manner of drinking PBS. Again, mediation analyses showed significant indirect effects, as follows. PNF reduced norms compared to both control and CAA, which were associated with fewer alcohol‐related consequences. CAA reduced dissonance compared to both control and PNF, which were associated with fewer alcohol‐related consequences. In contrast, PNF increased dissonance relative to control, which was associated with more alcohol‐related consequences. PNF and CAA both increased stopping/limiting PBS relative to control, but this change in PBS was not associated with more alcohol‐related consequences.

## DISCUSSION

The purpose of this manuscript was to examine a priori mechanisms of action specific to two very brief theory‐based interventions, PNF and CAA. Unlike PNF, which has been subject to extensive empirical study (see quantitative reviews by Dotson et al., 2015 and Miller et al., 2013), this is the first examination of how the novel alcohol CAA intervention might lead to the observed reductions in alcohol‐related consequences (Carey et al., 2025). The results of the mediation analyses suggest that the effects of the PNF and CAA interventions on alcohol use and related consequences work through both expected and unexpected pathways.

As hypothesized in *H1a* and *H1b*, PNF was effective in reducing normative perceptions of peer drinking relative to both the control and CAA groups; lower perceived norms, in turn, were associated with fewer drinks per week and fewer alcohol‐related consequences. This finding is consistent with a review (Reid & Carey, 2015), which identified reducing descriptive norms as the most consistently supported mediator of brief alcohol intervention for college drinkers. When PNF interventions successfully lower perceived drinking norms, especially when locally relevant normative comparisons are used, this change in perception typically results in a reduction in consumption and sometimes in consequences (Reid & Carey, 2015). The results of the present study suggest that even a very simple PNF intervention was effective in targeting its primary mechanism of action.

However, contrary to expectations in *H2a* and *H2b*, the interventions had opposite and unexpected effects on dissonance, which was proposed as a common mechanism of action. As predicted, the PNF increased cognitive dissonance compared to the control group. Our results indicated that presenting heavy drinkers with social norm information that challenges their assumptions about peer drinking did create measurable increases in dissonance, consistent with the dissonance‐based belief disconfirmation paradigm (Festinger, et al., 1956). However, this increased dissonance was associated with an *increase* in both drinking and alcohol‐related consequences. We note that overall PNF was associated with lower drinks per week in the parent study (Carey et al., 2025), and the present study confirms that this outcome is related to reduced drinking norms. However, to the extent that PNF also increases dissonance, this pathway has unintended adverse effects on drinking outcomes. Although using normative feedback to create dissonance or enhance discrepancy has been a hypothesized mechanism of PNF, it has rarely been tested (for an exception, see McNally et al., 2005). It is unclear under what conditions PNF might have a counterproductive dissonance‐increasing effect. Perhaps some of the heavy drinkers recruited into this study responded defensively, reducing the uncomfortable dissonance by discounting the new information rather than changing their drinking behavior to align with the newly presented norms. Indeed, health‐related information perceived as threatening to self‐image is likely to be avoided or discounted (e.g., Goldenberg & Arndt, 2008; Sherman et al., 2000).

In contrast and counter to expectation, CAA reduced levels of cognitive dissonance relative to both the control and PNF groups; however, this reduced dissonance was associated with fewer drinks per week and fewer alcohol‐related consequences. Although we had initially assumed that advocating to avoid the very consequences that one had experienced recently would create dissonance, the CAA prompt actually had two components—(a) explaining why drinking in a way that would avoid these consequences would be desirable (potentially dissonance‐inducing), and (b) recommending ways that college students could avoid consequences when they drink (potentially dissonance‐reducing). The second part of the CAA prompt could have functioned as a change plan, allowing the participants to articulate how they could drink in a way that reduces adverse effects, thereby reducing dissonance. Developing change plans in brief alcohol intervention has long been associated with better outcomes (Lee et al., 2010). Similarly, articulation of implementation intentions is also associated with reduced alcohol use (Malaguti et al., 2020; Norman et al., 2019). This post hoc explanation is, of course, speculative and subject to empirical validation.

We did not find support for CAA effects on attitudes (H3a), and attitude change was not a mechanism of action for either intervention being examined (H3b). Given that the CAA intervention was inspired by an attitude‐change paradigm, the lack of impact on attitudes toward heavy drinking was surprising. One explanation may be that the attitude measure we used was not sufficiently sensitive to change. Another explanation may be that the CAA‐inspired activity did not operate by reducing favorable attitudes toward heavy drinking. Attitude specificity is an important consideration for attitude‐behavioral correspondence (Ajzen & Timko, 1986). Perhaps the focus on reducing consequences had a more specific effect on attitudes toward experiencing alcohol‐related consequences. We note that the research on the use of CAA for smoking cessation, on which our CAA for alcohol reduction was based, did not measure change in attitudes (Simmons et al., 2013; Simmons & Brandon, 2007). Thus, other than reducing affect‐based dissonance, it is unclear what pathways may explain the ability of the CAA activity to reduce negative alcohol‐related consequences.

Finally, both interventions increased the use of stopping/limiting PBS in partial support of *H4a*; but this was not associated with changes in drinks per week nor alcohol‐related consequences, contrary to what we had anticipated in *H4b*. Neither condition caused any change in the Manner of Drinking or Serious Harm Reduction PBS. Although reviews have documented negative associations between overall PBS use and alcohol use and consequences (cf. Cox et al., 2024; Peterson et al., 2021), identifying the causal associations between PBS use and reduced drinking risk has been elusive. Many multicomponent alcohol interventions promote the use of PBS, but only a few studies have isolated the effect of promoting PBS use, and the relationship to alcohol outcomes has been mixed. For example, several studies have implemented streamlined interventions to promote PBS use (Martens et al., 2013; O'Donnell et al., 2019; Sugarman & Carey, 2009). All of these studies documented increased PBS use, but none found increased PBS to be associated with decreased drinking. On the other hand, Kenney et al. (2014) and LaBrie et al. (2015) delivered longer interventions that promoted PBS use via skill training or problem‐solving; similar to the previous studies, both interventions increased PBS use but did not change alcohol outcomes, relative to control conditions. However, these studies did find significant mediation whereby the intervention reduced drinking and consequences through increased PBS use. Thus, this literature suggests that there is not a clear relationship between changes in PBS use and changes in drinking or consequences. However, it is true that people who drink more frequently have more opportunities to use and endorse the kinds of protective strategies used while drinking (e.g., Braitman et al., 2015; Sugarman & Carey, 2007). In the present study, it is possible that some heavy drinking students responded to the CAA and PNF interventions by increasing their use of the one type of PBS—Stopping/Limiting strategies—that happen to be consistent with continuing to drink heavily (cf. Cox et al., 2024). It is also possible that they may have done so for different reasons—the CAA participants may have articulated plans to use protective strategies as an (unsuccessful) method of avoiding consequences, whereas the PNF participants may have engaged in Stopping/Limiting in an attempt to continue to drink even though they now knew they were drinking more than most peers.

These findings have several practical implications for designing and refining brief college drinking interventions. First, the results of the present work underscore the importance of mechanistic specificity: consistent with existing work (e.g., Reid & Carey, 2015), PNF interventions are effective largely due to their ability to reduce perceived drinking norms, while CAA appears to reduce consequences via a reduction, not increase, in dissonance. Moving forward, PNF could be modified to reduce dissonance and defensive responding—for example by incorporating brief writing‐based reflective exercises that encourage self‐reflection rather than defensiveness (e.g., Young & Neighbors, 2019). Additionally, screening procedures that identify students likely to respond defensively—for instance, those with particularly high baseline drinking or strong resistance to feedback (c.f., Leffingwell et al., 2007)—could trigger adaptive intervention strategies that mitigate counterproductive effects by providing students with either PNF or CAA based on how they respond. Finally, given that these interventions have different, sometimes opposing, mechanisms operating simultaneously, understanding these pathways can inform more tailored, mechanism‐focused strategies. It could also allow for the examination of a combined intervention that includes both PNF and CAA simultaneously. This could allow for adaptive, combined, intervention strategies that mitigate iatrogenic effects.

Several limitations of the current study should be noted. These include the potential impact of COVID‐19‐related recruitment effects, possible biases inherent in self‐reported data, and the relatively short follow‐up period, which may not adequately capture sustained or longer term changes in alcohol use or related negative outcomes. First, participant recruitment and follow‐up assessments occurred both prior to and following the onset of the COVID‐19 pandemic. The early stages of the pandemic, particularly during lockdowns, were associated with both increases and decreases in alcohol consumption, with patterns influenced by a range of contextual factors (Acuff et al., 2022). As a result, pandemic‐related variability could have impacted the theoretical mediators as well as the behavioral outcomes, which may have had some impact on the current study. Second, all drinking data (e.g., drinks per week and alcohol‐related consequences) were gathered through self‐reports, which may not perfectly align with objective assessments of alcohol consumption. Nonetheless, prior research has shown that self‐reported alcohol use is strongly correlated with objective indicators, suggesting minimal risk of biasing the findings of this study (Leffingwell et al., 2013). Third, the current sample of heavy drinkers evidenced heavier drinking than a recent sample of heavy drinkers from their respective campuses, which may limit the generalizability of these findings to other heavier drinking samples. Fourth, some measures, including attitudes toward heavy drinking, may not have been optimally aligned with the intended intervention targets (i.e., CAA), which focused on reducing alcohol‐related consequences rather than heavy drinking per se. In addition, dissonance was only assessed once, postintervention, which may have missed potentially more dynamic or nuanced processes that unfolded during the CAA intervention. Our selection of the dissonance thermometer was informed by prior work leveraging the foundations of the CAA paradigm in reducing behavioral addictions such as cigarette smoking (Simmons et al., 2004, 2013), but the current measure may not have captured the full range of psychological discomfort thought to be experienced when engaging with dissonance‐based paradigms. While our findings provide support for changes in cognitive dissonance as a mediator of both PNF and CAA (in opposite directions), we also recognize the ongoing scholarly debate about best practices in measuring dissonance (e.g., Devine et al., 2019; Vaidis & Bran, 2019). Future research might benefit from replicating these results using a broad range of dissonance measures to establish generalizability across operationalizations of the construct. Additionally, while we measured general PBS, we did not measure context‐specific PBS or PBS use and subsequent drinking at the day level, which may have contributed to PBS not being a supported mechanism of action for CAA. Finally, future work should endeavor to further evaluate the duration of these intervention effects with longer follow‐up assessment (e.g., 1‐year postbaseline) to determine how long they influence drinking and related consequences.

Taken together, these findings revealed that PNF and CAA work differently through distinctive mechanisms and highlight the complexity of intervention effects. We replicated the reduction in descriptive drinking norms after a PNF intervention but also found that PNF given to heavy college student drinkers increased dissonance and increased the use of Stopping/Limiting PBS, both of which lead to more negative alcohol outcomes. However, because the overall effect of PNF was to reduce drinks per week, the norms pathway appears to have precedence. The CAA intervention was associated with reduced dissonance but increased stopping/limiting PBS, with opposing effects and consequences. Because the overall effect of CAA was to reduce alcohol‐related consequences, the dissonance reduction mechanism appears to have preeminence. This work underscores the need for explicit empirical exploration of the underlying processes driving alcohol‐related interventions' efficacy.

## CONFLICT OF INTEREST STATEMENT

The authors have no conflicts of interest to report.

## Supporting information


Appendix S1

## Data Availability

The data that support the findings of this study are available on request from the corresponding author. The data are not publicly available due to privacy or ethical restrictions.
