# Supplementary material for: Mechanisms of change for two brief alcohol interventions: Testing theoretical mediators for counter attitudinal advocacy and personalized feedback intervention effects
Source: Alcohol Clin Exp Res (Hoboken). 2025 Dec 4;50(1):e70213. doi: 10.1111/acer.70213 (PMC12828102; doi:10.1111/acer.70213)
Supplement: Supplementary file 1 — Appendix S1 [file ACER-50-0-s001.docx]

Supplemental Tables 1-5

Supplemental Table 1

*Descriptive Norms and Attitude toward Heavy Drinking Step 1 and Step 2 Mediation Models for Alcohol Use and Alcohol-related Consequences*

| Outcome: Drinks Per Week | | | | | | | |
| --- | --- | --- | --- | --- | --- | --- | --- |
|  |  | Mediator | | | | | |
|  |  | Descriptive Norms | | | Attitudes Toward Heavy Drinking | | |
| Step 1 (a-path) | Predictor | b | Z | *e*^b^ | b | Z | *e*^b^ |
|  | **Intercept** | **1.711** | **37.710** | **5.534** | **1.273** | **9.780** | **--** |
|  | **Baseline mediator** | **0.035** | **12.450** | **1.036** | **0.549** | **13.750** | **--** |
|  | **Baseline Drinks Per Week** | **0.008** | **3.230** | **1.008** | **0.007** | **2.220** | **--** |
|  | **Site** | **0.103** | **3.010** | **1.108** | -0.004 | -0.070 | -- |
|  | **Male** | **0.141** | **3.960** | **1.151** | -0.005 | -0.100 | -- |
|  | **PNF (vs. Control)** | **-0.564** | **-13.110** | **0.569** | -0.048 | -0.800 | -- |
|  | CAA (vs. Control) | 0.027 | 0.740 | 1.027 | 0.061 | 1.050 | -- |
|  | **Time** | **-0.011** | **-2.440** | **0.989** | -0.009 | -1.250 | -- |
|  | lnalpha-dispersion | -4.100 | -- | -- | -- | -- | -- |
|  | random var(intercept) | 0.098 | -- | -- | 0.209 | -- | -- |
| Step 2 (b-path) | **Intercept** | **1.027** | **12.220** | **2.794** | **0.235** | **1.580** | **1.265** |
|  | Baseline mediator | -0.006 | -1.110 | 0.994 | 0.145 | 3.240 | 1.156 |
|  | **Change in Mediator** | **0.035** | **6.130** | **1.036** | **0.213** | **7.250** | **1.237** |
|  | **Baseline Drinks Per Week** | **0.049** | **7.490** | **1.051** | **0.047** | **8.070** | **1.048** |
|  | Site | -0.049 | -0.850 | 0.952 | 0.032 | 0.600 | 1.033 |
|  | **Male** | **0.146** | **2.420** | **1.157** | **0.218** | **3.800** | **1.243** |
|  | PNF (vs. Control) | -0.008 | -0.120 | 0.992 | **-0.170** | **-2.690** | **0.844** |
|  | CAA (vs. Control) | -0.053 | -0.830 | 0.948 | -0.067 | -1.090 | 0.935 |
|  | **Time** | **-0.020** | **-2.470** | **0.980** | **-0.021** | **-2.660** | **0.979** |
|  | lnalpha-dispersion | -1.222 | -- | -- | 0.120 | -- | -- |
|  | random var(intercept) | 0.212 | -- | -- | 0.031 | -- | -- |
| Outcome: Alcohol-related Consequences | | | | | | | |
| Step 1 (a-path) | Predictor | b | Z | *e*^b^ | b | Z | *e*^b^ |
|  | **Intercept** | **1.657** | **34.360** | **5.244** | **1.369** | **10.080** | **--** |
|  | **Baseline mediator** | **0.035** | **12.470** | **1.035** | **0.515** | **12.830** | **--** |
|  | **Baseline Drinks Per Week** | **0.014** | **5.680** | **1.014** | **0.021** | **6.910** | **--** |
|  | Baseline Consequences | 0.003 | 1.450 | 1.003 | -0.007 | -1.840 | -- |
|  | **Site** | **0.092** | **2.750** | **1.096** | 0.008 | 0.150 | -- |
|  | **Male** | **0.128** | **3.700** | **1.137** | -0.030 | -0.600 | -- |
|  | **PNF (vs. Control)** | **-0.547** | **-12.990** | **0.579** | -0.013 | -0.230 | -- |
|  | CAA (vs. Control) | 0.029 | 0.820 | 1.030 | 0.063 | 1.100 | -- |
|  | Time | -0.009 | -1.940 | 0.991 | -0.006 | -0.830 | -- |
|  | lnalpha-dispersion | -4.187 | -- | -- | -- | -- | -- |
|  | random var(intercept) | 0.090 | -- | -- | .202 | -- | -- |
| Step 2 (b-path) | **Intercept** | **0.841** | **8.450** | **2.319** | **0.620** | **3.510** | **1.859** |
|  | **Baseline mediator** | -0.002 | -0.290 | 0.998 | **0.136** | **2.850** | **1.145** |
|  | **Change in Mediator** | -0.011 | -1.830 | 0.989 | **-0.095** | **-2.940** | **0.910** |
|  | **Baseline Drinks Per Week** | **0.073** | **14.880** | **1.076** | **0.071** | **14.530** | **1.073** |
|  | **Baseline Consequences** | **0.056** | **12.140** | **1.057** | **0.055** | **11.960** | **1.057** |
|  | **Site** | **0.169** | **2.490** | **1.184** | **0.137** | **2.120** | **1.146** |
|  | **Male** | **-0.210** | **-2.980** | **0.810** | **-0.252** | **-3.790** | **0.777** |
|  | **PNF (vs. Control)** | -0.141 | -1.810 | 0.868 | -0.097 | -1.340 | 0.908 |
|  | **CAA (vs. Control)** | **-0.143** | **-2.010** | **0.867** | **-0.145** | **-2.060** | **0.865** |
|  | **Time** | **-0.028** | **-2.960** | **0.972** | **-0.028** | **-2.940** | **0.973** |
|  | lnalpha-dispersion | -0.925 | -- | -- | -0.929 | -- | -- |
|  | random var(intercept) | 0.274 | -- | -- | 0.270 | -- | -- |

*Note.* Bold text represents as significant effect.

Supplemental Table 2

*Cognitive Dissonance Step 1 and Step 2 Mediation Models for Alcohol Use and Alcohol-related Consequences*

| Outcome: Drinks Per Week | | | | |
| --- | --- | --- | --- | --- |
|  |  | Mediator | | |
|  |  | Dissonance | | |
| Step 1 (a-path) | Predictor | b | Z | *e*^b^ |
|  | **Intercept** | **1.905** | **15.240** | **6.716** |
|  | **Baseline Drinks Per Week** | **0.034** | **4.490** | **1.034** |
|  | Site | 0.148 | 1.440 | 1.160 |
|  | Male | -0.089 | -0.820 | 0.915 |
|  | **PNF (vs. Control)** | **0.320** | **3.000** | **1.377** |
|  | **CAA (vs. Control)** | **-0.519** | **-3.710** | **0.595** |
|  | lnalpha-dispersion | 1.715 | -- | -- |
| Step 2 (b-path) | **Intercept** | **1.268** | **15.380** | **3.552** |
|  | Post Intervention Dissonance | -0.004 | -1.690 | 0.996 |
|  | **Baseline Drinks Per Week** | **0.056** | **8.150** | **1.058** |
|  | Site | 0.026 | 0.440 | 1.026 |
|  | **Male** | **0.213** | **3.400** | **1.237** |
|  | **PNF (vs. Control)** | **-0.164** | **-2.440** | **0.849** |
|  | CAA (vs. Control) | -0.060 | -0.880 | 0.941 |
|  | Time | -0.022 | -2.690 | 0.978 |
|  | lnalpha-dispersion | 0.120 | -- | -- |
|  | random var(intercept) | 0.038 | -- | -- |
| Outcome: Alcohol-related Consequences | | | | |
| Step 1 (a-path) | Predictor | b | Z | *e*^b^ |
|  | **Intercept** | **1.411** | **9.780** | **4.102** |
|  | Baseline Drinks Per Week | -0.001 | -0.120 | 0.999 |
|  | **Baseline Consequences** | **0.070** | **9.990** | **1.073** |
|  | Site | -0.023 | -0.220 | 0.977 |
|  | Male | -0.038 | -0.370 | 0.963 |
|  | **PNF (vs. Control)** | **0.370** | **3.230** | **1.448** |
|  | **CAA (vs. Control)** | **-0.647** | **-4.940** | **0.524** |
|  | lnalpha-dispersion | 0.427 | -- | -- |
| Step 2 (b-path) | **Intercept** | **0.727** | **8.360** | **2.070** |
|  | **Post Intervention Dissonance** | **0.007** | **2.490** | **1.007** |
|  | **Baseline Drinks Per Week** | **0.071** | **14.820** | **1.073** |
|  | **Baseline Consequences** | **0.050** | **10.000** | **1.051** |
|  | **Site** | **0.139** | **2.150** | **1.149** |
|  | **Male** | **-0.247** | **-3.680** | **0.781** |
|  | PNF (vs. Control) | -0.123 | -1.660 | 0.885 |
|  | CAA (vs. Control) | -0.120 | -1.680 | 0.887 |
|  | **Time** | **-0.025** | **-2.630** | **0.975** |
|  | lnalpha-dispersion | 0.110 | -- | -- |
|  | random var(intercept) | 0.039 | -- | -- |

*Note.* Bold text represents as significant effect.

Supplemental Table 3

*Protective Behavioral Strategies Subscale Step 1 and Step 2 Mediation Models for Alcohol Use*

|  |  | Outcome | | |
| --- | --- | --- | --- | --- |
|  |  | Stopping/Liming | | |
| Step 1 (a-path) | Predictor | b | Z | *e*^b^ |
|  | Intercept | -0.135 | -1.140 | 0.873 |
|  | **Baseline Stopping/Limiting** | **0.473** | **16.390** | **1.605** |
|  | Changes in Stopping/Limiting | **--** | **--** | **--** |
|  | **Baseline Manner of Drinking** | **-0.123** | **-4.430** | **0.885** |
|  | **Changes in Manner of Drinking** | **0.399** | **20.910** | **1.490** |
|  | **Baseline Serious Harm Reduction** | **-0.093** | **-4.440** | **0.911** |
|  | **Changes in Serious Harm Reduction** | **0.234** | **17.170** | **1.264** |
|  | Baseline Drinks Per Week | -0.003 | -0.960 | 0.997 |
|  | **Site** | **0.171** | **3.600** | **1.187** |
|  | **Male** | **0.177** | **3.560** | **1.194** |
|  | **PNF (vs. Control)** | **0.114** | **2.070** | **1.120** |
|  | **CAA (vs. Control)** | **0.141** | **2.560** | **1.151** |
|  | Time | -0.013 | -1.810 | 0.987 |
|  | random var(intercept) | 0.170 | -- | -- |
|  |  | Manner of Drinking | | |
| Step 1 (a-path) | Predictor | b | Z | *e*^b^ |
|  | **Intercept** | **0.637** | **4.590** | **1.890** |
|  | **Baseline Stopping/Limiting** | **-0.276** | **-7.450** | **0.759** |
|  | **Changes in Stopping/Limiting** | **0.654** | **24.370** | **1.922** |
|  | **Baseline Manner of Drinking** | **0.401** | **12.210** | **1.493** |
|  | Changes in Manner of Drinking | -- | -- | -- |
|  | Baseline Serious Harm Reduction | -0.033 | -1.240 | 0.967 |
|  | **Changes in Serious Harm Reduction** | **0.120** | **5.820** | **1.128** |
|  | Baseline Drinks Per Week | -0.004 | -1.160 | 0.996 |
|  | **Site** | **-0.121** | **-2.170** | **0.886** |
|  | **Male** | **-0.202** | **-3.400** | **0.817** |
|  | PNF (vs. Control) | -0.008 | -0.130 | 0.992 |
|  | CAA (vs. Control) | -0.076 | -1.170 | 0.927 |
|  | **Time** | **0.024** | **2.500** | **1.024** |
|  | random var(intercept) | 0.212 | -- | -- |
|  |  | Serious Harm Reduction | | |
| Step 1 (a-path) | Predictor | b | Z | *e*^b^ |
|  | **Intercept** | **0.923** | **5.010** | **2.516** |
|  | **Baseline Stopping/Limiting** | **-0.299** | **-6.030** | **0.742** |
|  | **Changes in Stopping/Limiting** | **0.709** | **16.970** | **2.031** |
|  | **Baseline Manner of Drinking** | **-0.107** | **-2.210** | **0.899** |
|  | **Changes in Manner of Drinking** | **0.226** | **5.760** | **1.253** |
|  | **Baseline Serious Harm Reduction** | **0.371** | **11.140** | **1.449** |
|  | Changes in Serious Harm Reduction | -- | -- | -- |
|  | **Baseline Drinks Per Week** | **0.011** | **1.990** | **1.011** |
|  | Site | -0.094 | -1.210 | 0.910 |
|  | **Male** | **-0.263** | **-3.250** | **0.769** |
|  | PNF (vs. Control) | -0.058 | -0.650 | 0.943 |
|  | CAA (vs. Control) | -0.087 | -0.960 | 0.917 |
|  | Time | -0.024 | -1.830 | 0.977 |
|  | random var(intercept) | 0.384 | -- | -- |
|  |  | Alcohol Use | | |
| Step 2 (b-path) | Predictor | b | Z | *e*^b^ |
|  | **Intercept** | **0.801** | **5.070** | **2.227** |
|  | **Baseline Stopping/Limiting** | **-0.104** | **-2.420** | **0.901** |
|  | Changes in Stopping/Limiting | 0.058 | 1.760 | 1.060 |
|  | Baseline Manner of Drinking | -0.047 | -1.380 | 0.954 |
|  | Changes in Manner of Drinking | 0.031 | 1.170 | 1.032 |
|  | Baseline Serious Harm Reduction | -0.045 | -1.750 | 0.956 |
|  | **Changes in Serious Harm Reduction** | **0.207** | **10.570** | **1.230** |
|  | **Baseline Drinks Per Week** | **0.053** | **8.040** | **1.054** |
|  | Site | 0.018 | 0.330 | 1.018 |
|  | **Male** | **0.313** | **4.880** | **1.367** |
|  | **PNF (vs. Control)** | **-0.209** | **-3.230** | **0.811** |
|  | CAA (vs. Control) | -0.054 | -0.840 | 0.947 |
|  | **Time** | **-0.021** | **-2.660** | **0.979** |
|  | lnalpha-dispersion | -1.476 | -- | -- |
|  | random var(intercept) | 0.234 | -- | -- |

*Note.* Bold text represents as significant effect.

Supplemental Table 4

*Protective Behavioral Strategies Subscale Step 1 and Step 2 Mediation Models for Alcohol-related Consequences*

|  |  | Outcome | | |
| --- | --- | --- | --- | --- |
|  |  | Stopping/Liming | | |
| Step 1 (a-path) | Predictor | b | Z | *e*^b^ |
|  | Intercept | -0.170 | -1.390 | 0.843 |
|  | **Baseline Stopping/Limiting** | **0.477** | **16.690** | **1.611** |
|  | Changes in Stopping/Limiting | -- | -- | -- |
|  | **Baseline Manner of Drinking** | **-0.122** | **-4.430** | **0.885** |
|  | **Changes in Manner of Drinking** | **0.401** | **21.100** | **1.494** |
|  | **Baseline Serious Harm Reduction** | **-0.094** | **-4.480** | **0.910** |
|  | **Changes in Serious Harm Reduction** | **0.230** | **16.050** | **1.259** |
|  | Drinks Per Week | 0.002 | 0.510 | 1.002 |
|  | Baseline Alcohol-related Consequences | 0.000 | -0.100 | 1.000 |
|  | **Site** | **0.166** | **3.460** | **1.181** |
|  | **Male** | **0.162** | **3.230** | **1.176** |
|  | **PNF (vs. Control)** | **0.123** | **2.240** | **1.131** |
|  | **CAA (vs. Control)** | **0.143** | **2.580** | **1.153** |
|  | Time | -0.013 | -1.900 | 0.987 |
|  | random var(intercept) | 0.171 | -- | -- |
|  |  | Manner of Drinking | | |
| Step 1 (a-path) | Predictor | b | Z | *e*^b^ |
|  | **Intercept** | **0.581** | **4.030** | **1.788** |
|  | **Baseline Stopping/Limiting** | **-0.292** | **-7.940** | **0.746** |
|  | **Changes in Stopping/Limiting** | **0.655** | **24.580** | **1.926** |
|  | **Baseline Manner of Drinking** | **0.413** | **13.420** | **1.511** |
|  | Changes in Manner of Drinking | -- | -- | -- |
|  | Baseline Serious Harm Reduction | -0.039 | -1.490 | 0.962 |
|  | **Changes in Serious Harm Reduction** | **0.129** | **6.020** | **1.137** |
|  | Drinks Per Week | -0.007 | -1.820 | 0.993 |
|  | Baseline Alcohol-related Consequences | 0.007 | 1.600 | 1.007 |
|  | **Site** | **-0.134** | **-2.290** | **0.875** |
|  | **Male** | **-0.199** | **-3.320** | **0.820** |
|  | PNF (vs. Control) | -0.029 | -0.460 | 0.971 |
|  | CAA (vs. Control) | -0.073 | -1.130 | 0.929 |
|  | **Time** | **0.023** | **2.450** | **1.024** |
|  | random var(intercept) | 0.212 | -- | -- |
|  |  | Serious Harm Reduction | | |
| Step 1 (a-path) | Predictor | b | Z | *e*^b^ |
|  | **Intercept** | **0.846** | **4.530** | **2.330** |
|  | **Baseline Stopping/Limiting** | **-0.249** | **-5.140** | **0.779** |
|  | **Changes in Stopping/Limiting** | **0.658** | **15.920** | **1.932** |
|  | **Baseline Manner of Drinking** | **-0.099** | **-2.170** | **0.906** |
|  | **Changes in Manner of Drinking** | **0.230** | **6.020** | **1.258** |
|  | **Baseline Serious Harm Reduction** | **0.355** | **11.190** | **1.426** |
|  | Changes in Serious Harm Reduction | -- | -- | -- |
|  | **Drinks Per Week** | **0.051** | **9.510** | **1.053** |
|  | **Baseline Alcohol-related Consequences** | **-0.015** | **-3.150** | **0.985** |
|  | Site | -0.058 | -0.780 | 0.943 |
|  | **Male** | **-0.362** | **-4.740** | **0.696** |
|  | PNF (vs. Control) | 0.015 | 0.180 | 1.015 |
|  | CAA (vs. Control) | -0.078 | -0.900 | 0.925 |
|  | Time | -0.016 | -1.260 | 0.984 |
|  | random var(intercept) | 0.330 | -- | -- |
|  |  | Alcohol-related Consequences | | |
| Step 2 (b-path) | Predictor | b | Z | *e*^b^ |
|  | **Intercept** | **0.649** | **3.830** | **1.913** |
|  | Baseline Stopping/Limiting | -0.052 | -1.180 | 0.950 |
|  | **Changes in Stopping/Limiting** | **0.077** | **2.150** | **1.080** |
|  | Baseline Manner of Drinking | -0.043 | -1.220 | 0.958 |
|  | Changes in Manner of Drinking | 0.010 | 0.370 | 1.010 |
|  | **Baseline Serious Harm Reduction** | **-0.117** | **-4.080** | **0.890** |
|  | **Changes in Serious Harm Reduction** | **0.171** | **7.950** | **1.186** |
|  | **Drinks Per Week** | **0.060** | **13.720** | **1.062** |
|  | **Baseline Alcohol-related Consequences** | **0.058** | **12.530** | **1.059** |
|  | Site | 0.126 | 1.950 | 1.134 |
|  | **Male** | **-0.187** | **-2.690** | **0.829** |
|  | **PNF (vs. Control)** | **-0.147** | **-2.020** | **0.863** |
|  | **CAA (vs. Control)** | **-0.159** | **-2.200** | **0.853** |
|  | **Time** | **-0.024** | **-2.530** | **0.976** |
|  | lnalpha-dispersion | -1.095 | -- | -- |
|  | random var(intercept) | 0.288 | -- | -- |

*Note.* Bold text represents as significant effect.

Supplemental Table 5

*Summary Table of Key Findings by Hypothesis*

| **Hypothesis** | **Key Prediction** | **Outcome/Measure** | **Finding** |
| --- | --- | --- | --- |
| H1a | PNF reduces perceived peer drinking norms | Perceived descriptive norms | Supported: PNF reduced norms vs Control & CAA |
| H1b | Reduced norms mediate lower drinks & consequences | Drinks per week, alcohol-related consequences | Supported: Mediation significant |
| H2a | Interventions increase cognitive dissonance | Post-intervention cognitive dissonance | Mixed: PNF ↑ dissonance; CAA ↓ dissonance |
| H2b | Dissonance mediates effects on drinks & consequences | Drinks per week, consequences | Mixed: PNF ↑ dissonance ↑ drinking, CAA ↓ dissonance ↓ drinking |
| H3a | CAA reduces positive attitudes toward heavy drinking | Attitudes toward heavy drinking | Not supported: No change in attitudes |
| H3b | Attitude change mediates reduction in drinks & consequences | Drinks per week, consequences | Not supported |
| H4a | CAA increases use of protective behavioral strategies (PBS) | PBS Subscales | Partially supported: Stopping/Limiting PBS use ↑ in both interventions |
| H4b | PBS mediates reductions in drinks & consequences | Drinks per week, consequences | Not supported: Stopping/Limiting PBS use ↑ but not linked to outcomes |
